# Meniscal Repair in Athletes: Functional Outcomes and Return to Sport

**DOI:** 10.7759/cureus.101685

**Published:** 2026-01-16

**Authors:** Oussama Lassioued, Julien Amzallag, Hatem Abbassi, Hakami Fariborz, Tarek Naanaa

**Affiliations:** 1 Orthopedic Surgery and Trauma, Hospital Center of Provins, Provins, FRA; 2 Orthopedic Knee Surgery, Clinique Drouot, Paris, FRA; 3 Orthopedic Knee Surgery, Clinique Maussins-Nollet, Paris, FRA; 4 Surgery, Hospital Center of Provins, Provins, FRA

**Keywords:** athletes, functional outcomes, ikdc, meniscal repair, return to sport

## Abstract

Introduction: Arthroscopic meniscal repair is the preferred treatment for meniscal tears in athletes, but return to sport (RTS) after surgery remains a significant concern. This study aimed to evaluate factors influencing healing, functional outcomes, and RTS after isolated meniscal repair.

Methods: A retrospective study was conducted. Inclusion criteria (athletes < 50 years of age, isolated meniscal repair without concomitant anterior cruciate ligament (ACL) injury, ≥12 months of follow-up) yielded a final cohort of 53 patients. Evaluation was based on demographic, surgical, and clinical data, including preoperative and postoperative subjective International Knee Documentation Committee (IKDC) and Tegner functional scores.

Results: At a mean follow-up of 21 ± 8.5 months, functional improvement was observed. The mean subjective IKDC score improved from 48.5 preoperatively to 76.2 postoperatively. The RTS rate was 46 (86.8%), with 35 (66%) of the total cohort returning to their pre-injury sport level. The mean time to return to running and training was 6.1 ± 2.8 months. Factors associated with positive outcomes included surgery within three months of injury (better Tegner score, p = 0.029), age < 20 years (better Tegner score, p = 0.020), non-smoking status (better IKDC score, p = 0.020), a vertical tear pattern (better IKDC and Tegner scores, p = 0.040 and p = 0.010, respectively), and a high preoperative functional status (IKDC score > 40 and Tegner score > 5).

Conclusion: Meniscal repair is a preferred option for young, active athletes, offering good short- to medium-term outcomes and a high RTS rate. These findings suggest the need for individualized assessment to optimize recovery and establish a realistic prognosis.

## Introduction

Meniscal tears are a prevalent joint injury, with an estimated 60-70 new cases per 100,000 people annually, primarily affecting young, active individuals and athletes [1]. The menisci play a crucial role in the knee's physiology. They provide joint congruence, distribute load, absorb shock, stabilize the knee, lubricate the joint, and contribute to proprioception. Preserving their integrity is vital for the long-term health of the knee [2,3]. Historically, meniscectomy (partial or total) has been the standard treatment, providing quick symptom relief and a rapid return to sport (RTS) [1,4,5]. However, numerous studies have shown that this approach leads to deleterious long-term effects, including the frequent development of early and severe osteoarthritis [6,7]. This understanding has led to a significant shift toward meniscal preservation as the preferred approach whenever possible. Arthroscopic meniscal repair represents a conservative strategy that aims to heal the lesion and restore the meniscus's original anatomy and function [8-10].

Nevertheless, the success of a meniscal repair is not guaranteed. It depends on a complex and sometimes unpredictable biological healing process influenced by numerous factors. The recovery and rehabilitation are also more demanding and lengthy compared to a meniscectomy, which delays the RTS [11-13]. For athletes, the primary concern is the timeline and likelihood of returning to their previous performance level [14,15]. Given this, it is crucial to understand the factors that determine the success of an isolated meniscal repair in this population. The primary objective of this study was to thoroughly evaluate the factors influencing meniscal healing, functional outcomes, and RTS in athletes. A secondary objective was to review and discuss the criteria for resuming sports based on existing literature.

## Materials and methods

This retrospective, observational study included patients who underwent surgery from March 2021 to June 2023. The study included patients younger than 50 who were active in sports before arthroscopic meniscal repair and who had at least 12 months of postoperative follow-up. Patients were excluded if they had irreparable degenerative-type tears, specific injuries such as meniscal root or red zone avulsion of the medial meniscus posterior horn (RAMP) lesions, or associated ligament damage, including an anterior cruciate ligament (ACL) rupture. Additionally, the analysis excluded individuals with a history of surgery on the same knee, significant cartilage damage, pre-existing osteoarthritis, or those who were lost to follow-up.

Preoperative and postoperative clinical assessment

Patient data were extracted from medical records using a standardized form that captured demographic information (age, gender, affected side, and smoking status) and the mechanism of injury. Preoperative sports activity was evaluated with the Tegner Activity Scale [16,17]. Clinical data collected prior to surgery included primary functional symptoms (such as pain, joint blockage, and swelling), the subjective International Knee Documentation Committee (IKDC) score [8,18], and findings from a physical examination assessing joint mobility and clinical signs of meniscal syndrome using provocative tests, including the McMurray, Thessaly, and Apley's Grind tests. Clinical evaluations were performed at least 12 months postoperatively. Return to sport (RTS) was defined as the resumption of any athletic activity, while return to the pre-injury level was defined as achieving a postoperative Tegner score equal to the pre-injury score. During follow-up consultations, systematic clinical examinations were conducted to identify objective indicators of healing failure, including joint space tenderness, effusion, a positive McMurray test, or joint blockage, as outlined by Barrett [19] and Miao et al. [20]. A retrospective review of medical records was undertaken to identify immediate or short-term complications, with particular attention to vascular, neurological, or septic events. At each visit, the subjective IKDC score was recalculated for functional assessment, and key RTS data were recorded, including the timeline for resuming running and training and the postoperative Tegner activity level.

Preoperative radiological assessment

Magnetic resonance imaging (MRI), the most sensitive non-invasive examination for meniscal pathology [7,21], was used in conjunction with radiography. The diagnosis of a meniscal tear was confirmed by either identifying an intrameniscal hypersignal or by observing specific morphological and positional changes of the meniscus, such as a truncated, inverted, or globular appearance; a posterior double cruciate ligament appearance; an anterior double-horned appearance; or meniscal detachment, extrusion, or the "ghost meniscus sign" [22].

Imaging analysis defined the precise lesion location (medial/lateral; anterior/middle/posterior segment) and confirmed the absence of other significant pathologies (e.g., severe chondropathy, ligament injuries) as per the exclusion criteria. A postoperative MRI was not systematically performed to assess meniscal healing.

Surgical technique

The analysis of operative reports, along with any available intraoperative arthroscopic videos, was conducted first to confirm the absence of associated lesions in the ligaments, capsule, synovium, or articular surface. The meniscal lesion was precisely characterized according to the International Society of Arthroscopy, Knee Surgery and Orthopaedic Sports Medicine (ISAKOS) classification [23], detailing its type, depth, location by zone and segment, and degenerative status.

**Figure 1:**
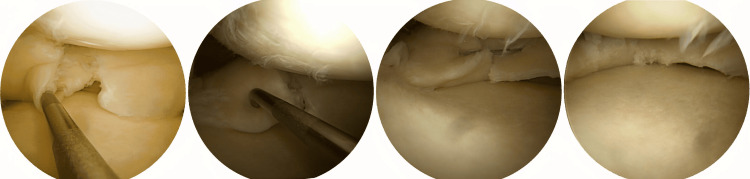
All-Inside repair of a radial tear proximal to the posterior root of the medial meniscus

**Figure 2:**
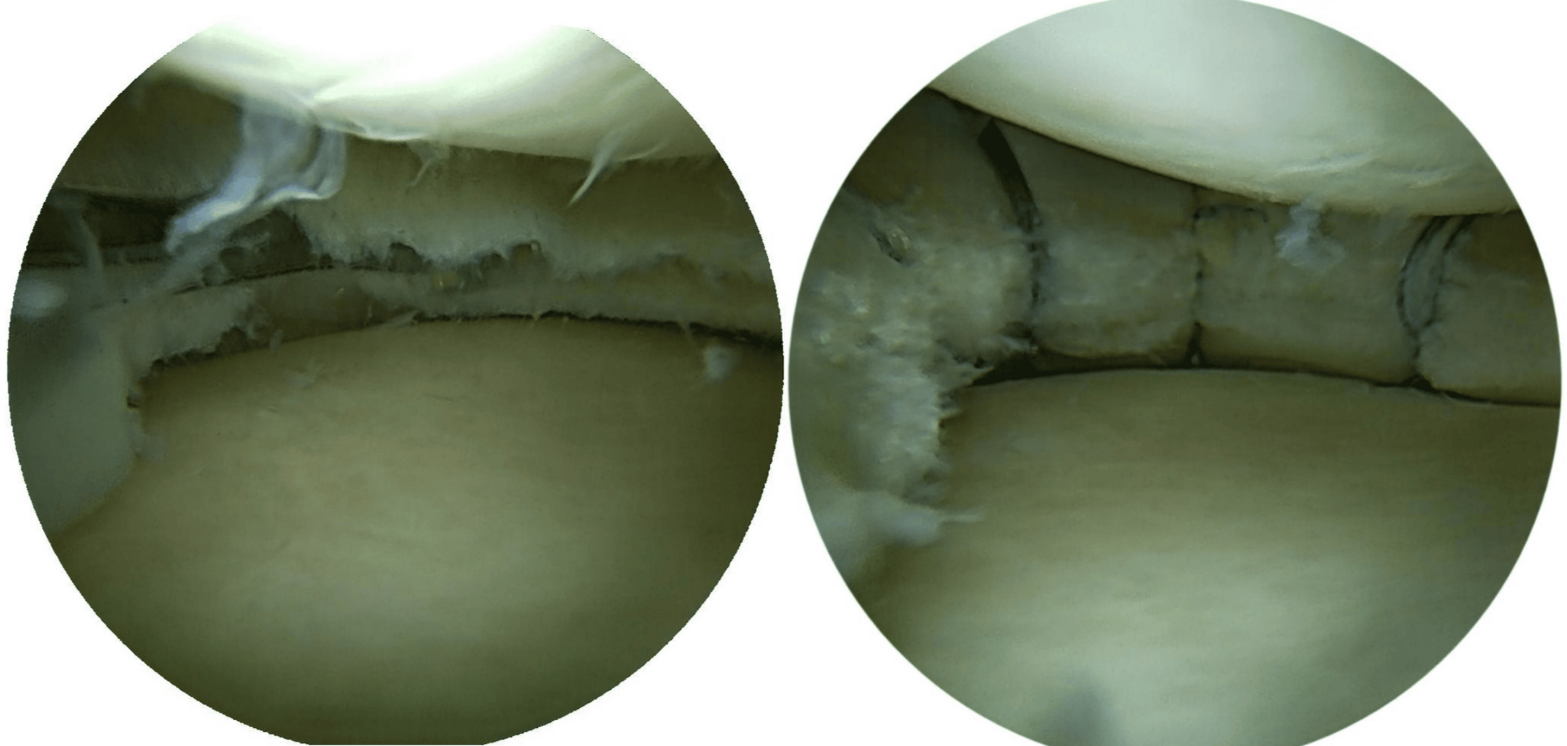
Repair of a horizontal meniscal tear using the Scorpion forceps

Various suturing methods were employed, ranging from second-generation methods to the most modern devices, including the All-Inside (AI) technique using Smith & Nephew's Fast-Fix kits, Arthrex's FiberStitch (Figure 1), or the Scorpion forceps (Figure 2); the Outside-In (OI) technique with cannulated needles and Polydioxanone Suture (PDS) thread; and the Inside-Out (IO) technique with PDS needles or an Arthrex Protector Meniscus Suturing Set, used either alone or in combination.

Pie-crusting of the medial collateral ligament was frequently performed for medial meniscus injuries. All repairs were performed after careful debridement of the lesion's edges with a shaver or rasp, and stimulation of the intercondylar notch (marrow venting) was systematically performed. No other biological augmentation was used. A final systematic assessment of the construct's stability was always carried out.

Postoperative rehabilitation

Postoperatively, patients adhered to a phased rehabilitation protocol with specific objectives and progressive restrictions on joint mobilization and weight-bearing. For all types of repairs, the knee range of motion (ROM) was limited to 90° of flexion during the first month.

The weight-bearing protocol was stratified based on the stability of the repaired lesion, defined intraoperatively as the absence of displacement under arthroscopic probing. Unstable tears, including radial, bucket-handle, oblique (parrot's beak), complex tears, and meniscal flaps, required strict touchdown weight-bearing for 4-6 weeks. In contrast, lesions considered stable, including vertical longitudinal tears and horizontal cleavage tears, were typically permitted partial weight-bearing as tolerated. Generally, patients were cleared to resume normal daily activities after approximately three months.

Data and statistical analysis

Data were captured and analyzed using IBM SPSS Statistics for Windows, Version 25.0 (Released 2017; IBM Corp., Armonk, NY, USA). Continuous quantitative variables were expressed as mean ± standard deviation (SD) and range (minimum-maximum), while categorical variables were presented as counts (n) and relative frequencies (%). The normality of data distribution was confirmed using the Kolmogorov-Smirnov and Shapiro-Wilk tests for each variable. To control for Type I error during multiple pairwise comparisons, a Bonferroni correction was applied.

An analytical study was conducted to evaluate the influence of various factors on final functional outcomes and the likelihood of returning to sport. For comparisons of categorical variables, Pearson's chi-square test or Fisher's exact test (for expected cell counts below 5) was used. To compare the means of a continuous variable between two independent groups, the Student's t-test was employed for normal distributions, while the non-parametric Mann-Whitney test was used otherwise. For comparisons involving more than two groups, analysis of variance (ANOVA) or its non-parametric equivalent, the Kruskal-Wallis test, was performed. Test statistic values (t, F, or chi-square) were reported alongside p-values. When global tests yielded significant results, post-hoc pairwise comparisons were conducted to identify specific intergroup differences. In all statistical tests, the significance level was set at p < 0.05. A post hoc power analysis was performed to assess the statistical power of the sample size for the primary functional outcome (subjective IKDC).

## Results

**Figure 3:**
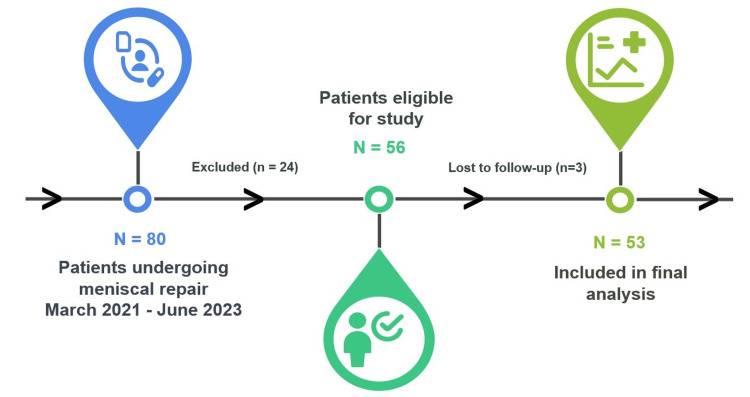
Flow diagram of the patient selection process

A total of 53 patients were included, with a mean follow-up of 21.0 ± 8.5 months (12-39). Figure 3 illustrates the study timeline.

As described in Table 1, the cohort was predominantly male (47, 88.7%), with a sex ratio (M/F) of 7.83. The mean age at the time of surgery was 28.0 ± 5.8 years (19-49). Thirty-one cases (58.5%) affected the right knee. Ten patients (18.8%) were identified as active smokers.

**Table 1:** Preoperative epidemiological, clinical, and radiological data (N = 53) IKDC: International Knee Documentation Committee, MVA: motor vehicle accident.

Demographics	
Age	28.0 ± 5.8 (range: 19-49)
Male	47 (88.7%)
Female	6 (11.3%)
Smokers	10 (18.8%)
Tegner Activity Level (pore-injury)	
Level 4	1 (1.9%)
Level 5	13 (24.5%)
Level 7	3 (5.7%)
Level 8	32 (60.4%)
Level 9	4 (7.5%)
Etiologies	
Sports accident	33 (62.3%)
Domestic accident	12 (22.6%)
MVA/work accident	4 (7.5%)
Other	4 (7.5%)
Clinical symptoms	
Pain	25 (47.2%)
Joint locking	18 (34.0%)
Giving way	12 (22.6%)
Cracking	10 (18.9%)
Swelling	7 (13.2%)
IKDC score	
<40	4 (7.5%)
40-49	30 (56.6%)
50-59	19 (35.8%)
MRI assessment	
Medial meniscus	35 (66.0%)
Lateral meniscus	18 (34.0%)
Posterior segment	35 (66.0%)
Middle segment	23 (43.4%)
Anterior segment	8 (15.1%)

The study population was athletically active, with 39 (73.6%) participants presenting with a preoperative Tegner score greater than 5. A sports-related accident was the primary cause of injury in 33 cases (62.3%).

The most common preoperative functional symptoms were pain (25, 47.2%) and joint locking (18, 34.0%), as presented in Table 1. On clinical examination, eight patients presented with a flexion deficit. The Thessaly test was the most frequently positive meniscal sign (38, 71.7%), followed by the Grinding test (34, 64.2%) and the McMurray test (30, 56.6%). The cohort's mean preoperative subjective IKDC score was 48.5 ± 5.2 (38-59). 

Initial radiological assessment, including standard X-rays and MRI, was performed for all 53 patients. MRI analysis identified a signal abnormality indicative of a tear in 49 (92.5%) cases and morphological or positional meniscal changes in 41 (77.4%) cases.

The medial meniscus was the most commonly involved (35, 66.0%), with lesions primarily located in the posterior segment (35, 66.0%). Detailed radiological information is provided in Table 1. In 13 patients, the tear extended across both the posterior and middle meniscal segments.

Operative and rehabilitation data

The time from symptom onset to surgical intervention varied among patients. Twenty-three patients (43.4%) underwent surgery within three months of symptom onset, while 20 patients (37.7%) underwent surgery between three and six months after symptom onset. Additionally, a delay of 6-12 months was observed in 10 patients (18.9%).

Consistent with the preoperative MRI findings, intraoperative arthroscopic exploration revealed no associated ligamentous, capsular, synovial, or articular surface lesions. The procedure confirmed medial meniscus involvement in 35 cases (66.0%) and lateral meniscus involvement in 18 cases (34.0%), with no bilateral lesions observed. Meniscal tear locations matched MRI findings, specified by ISAKOS classification (Table 2).

**Table 2:** Meniscus tears characteristics according to ISAKOS classification ISAKOS: International Society of Arthroscopy, Knee Surgery and Orthopaedic Sports Medicine.

Depth	
Partial	28 (52.8%)
Complete	25 (47.2%)
Rim zone (circumferential location)	
Zone 1 (red-red)	8 (15.1%)
Zone 2 (red-white)	37 (69.8%)
Zone 3 (white-white)	8 (15.1%)
Segment (radial location)	
Anterior segment	8 (15.1%)
Middle segment	23 (43.4%)
Posterior segment	35 (66.0%)
Tear type	
Vertical longitudinal	28 (52.8%)
Vertical flap	5 (9.4%)
Horizontal	7 (13.2%)
Horizontal flap	3 (5.7%)
Radial	5 (9.4%)
Complex	5 (9.4%)
Degenerative aspect	
Yes	12 (22.6%)
No	41 (77.4%)

The arthroscopic repairs were performed using a combination of techniques tailored to each lesion's configuration. Of the 53 patients, 17 (32.1%) required at least two combined techniques. The AI technique was the most frequently used (35 cases, 66.0%), followed by the IO technique (25 cases, 47.2%) and the OI technique (10 cases, 18.8%).

All patients followed the standardized postoperative rehabilitation protocol. Full weight-bearing was permitted at 30 days post-surgery for 35 patients (66.0%), whereas the remaining 18 patients (34.0%) were authorized to bear weight after 45 days.

Clinical and return to sport outcomes

At the final follow-up, the postoperative clinical examination was unremarkable in 43 patients (81.1%). Ten patients (18.9%) exhibited at least one clinical sign suggestive of healing failure, as per the Barrett and Miao criteria. Among this group, these signs were often associated in the same patient: joint line tenderness was present in eight cases (80%), joint effusion in six cases (60%), a positive McMurray test in five cases (50%), and joint locking in three cases (30%).

A limitation in the knee ROM was noted in some patients. The mean flexion was 125° ± 11.5° (90°-135°). Of the 10 patients with signs of healing failure, seven had limited flexion (<110°), whereas all other patients achieved flexion of 120° or greater. No patient presented with a residual deficit in extension or any immediate or short-term vascular, nervous, or septic complications. Functional improvement was observed following the surgical procedure. The average IKDC score increased from 48.5 ± 5.2 (38-59) preoperatively to 76.2 ± 10.1 (52-92) postoperatively. A post-hoc power analysis confirmed that a sample size of 53 patients provided >99% statistical power to detect this significant functional improvement (alpha = 0.05).

**Figure 4:**
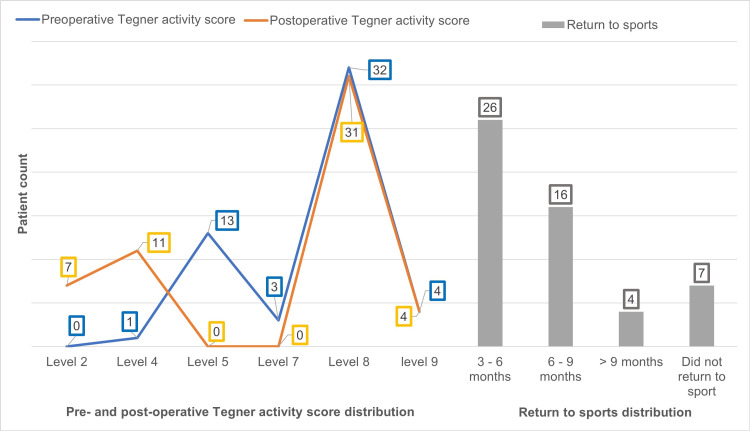
Postoperative Tegner activity level and return to sport outcomes

Regarding the RTS, 46 patients (86.8%) resumed sports activities, including running and training. Among them, 35 patients (representing 76.1% of those who returned to sport and 66% of the total cohort) regained their pre-injury level and maintained the same Tegner score. The 11 patients who did not return to their pre-injury level mainly had lower preoperative Tegner scores (≤5). The mean Tegner score for the cohort declined from 7.2 ± 1.3 to 6.45 ± 2.3 postoperatively. The mean time to return to running and training was 6.1 ± 2.8 months (3-13.5), with a median of 4.5 months (Figure 4).

A detailed analysis of patient- and surgery-related factors (Table 3) showed several factors associated with postoperative functional outcomes. Younger age (< 20 years), shorter time to surgery (< 3 months), and higher preoperative activity level (Tegner > 5) were associated with significantly higher postoperative Tegner scores. Conversely, smoking was associated with a significantly lower postoperative IKDC score, and a low preoperative IKDC score (< 40) was associated with significantly lower postoperative IKDC and Tegner scores. Regarding lesion characteristics, vertical longitudinal tears were associated with significantly higher IKDC and Tegner scores than other tear types, particularly complex lesions.

**Table 3:** Analysis of patient- and surgery-related factors associated with postoperative outcomes The p-value is presented with the corresponding test statistic in parentheses (t-value for Student’s t-test; F-value for ANOVA) Differences between two independent groups (sex, side, meniscus, smoking, full weight-bearing, pre-op Tegner) were analyzed using the Student’s t-test. Comparisons involving more than two groups (age, time to surgery, topography, tear type, pre-op IKDC) were analyzed using one-way ANOVA. Different superscript letters (a, b, c) within the same column indicate a statistically significant difference (p < 0.05) between specific subgroups.

Factor	N (%)	Mean post-op IKDC ± SD	p-value (Stat)	Mean post-op Tegner ± SD	p-value (Stat)
Age					
<20 years	5 (9.4%)	78.4 ± 8.5	0.659 (F = 0.42)	6.55 ± 0.8ᵃ	0.020 (F = 4.22)
20-30 years	15 (28.3%)	76.4 ± 10.2		6.45 ± 1.2ᵇ	
>30 years	33 (62.3%)	75.85 ± 11.4		6.44 ± 1.6ᵇ	
Sex					
Male	47 (88.7%)	76.3 ± 10.8	0.804 (t = 0.25)	6.46 ± 1.7	0.881 (t = 0.15)
Female	6 (11.3%)	75.4 ± 12.1		6.35 ± 1.5	
Side					
Right	31 (58.5%)	77.1 ± 11.0	0.457 (t = 0.75)	6.46 ± 1.8	0.858 (t = 0.18)
Left	22 (41.5%)	74.9 ± 10.5		6.35 ± 1.6	
Time to surgery					
<3 months	23 (43.4%)	78.1 ± 9.9	0.176 (F = 1.80)	7.15 ± 1.4 ᵃ	0.029 (F = 3.85)
3-6 months	20 (37.7%)	76.0 ± 11.2		6.35 ± 1.7 ᵇ	
>6 months	10 (18.9%)	73.8 ± 12.5		5.40 ± 1.9 ᶜ	
Meniscus					
Lateral	18 (34.0%)	74.48 ± 11.8	0.416 (t = 0.82)	6.35 ± 1.8	0.781 (t = 0.28)
Medial	35 (66.0%)	76.91 ± 10.4		6.50 ± 1.7	
Smoking					
Yes	10 (18.9%)	68.0 ± 12.4	0.020 (t = 2.38)	5.75 ± 1.8	0.260 (t = 1.14)
No	43 (81.1%)	78.1 ± 11.2		6.61 ± 2.1	
Topography (rim)					
Zone 1 (red-red)	8 (15.1%)	77.75 ± 9.8	0.887 (F = 0.12)	6.83 ± 1.5	0.640 (F = 0.45)
Zone 2 (red-white)	37 (69.8%)	75.97 ± 10.5		6.44 ± 2.0	
Zone 3 (white-white)	8 (15.1%)	75.63 ± 11.1		6.14 ± 1.9	
Tear type					
Vertical longitudinal (incl. flap)	33 (62.3%)	81.0 ± 9.5ᵃ	0.040 (F=2.98)	6.84 ± 1.4ᵃ	0.010 (F = 4.15)
Horizontal (incl. flap)	10 (18.9%)	70.0 ± 12.1ᵇ		6.05 ± 1.8ᵇ	
Radial	5 (9.4%)	67.2 ± 13.5ᵇ		5.60 ± 2.1ᵇᶜ	
Complex	5 (9.4%)	63.4 ± 14.0 ᶜ		5.07 ± 2.2 ᶜ	
Full weight-bearing					
<30 days	35 (66.0%)	76.63 ± 10.1	0.519 (t = 0.65)	7.15 ± 1.6	0.984 (t = 0.02)
>30 days	18 (34.0%)	74.72 ± 10.9		7.14 ± 1.9	
Pre-op Tegner					
≤5	14 (26.4%)	74.85 ± 11.5	0.145 (t=1.48)	6.07 ± 2.2	0.031 (t=2.21)
>5	39 (73.6%)	79.79 ± 9.8		7.49 ± 1.5	
Pre-op IKDC					
<40	4 (7.5%)	70.0 ± 13.2ᵇ	0.015 (F = 4.56)	5.65 ± 2.4ᵇ	0.021 (F = 4.18)
40-49	30 (56.6%)	76.0 ± 10.4ᵃ		6.40 ± 1.7ᵃ	
50-59	19 (35.8%)	78.0 ± 8.9ᵃ		6.75 ± 1.5ᵃ	

## Discussion

A significant concern for patients with regular sports activity who are candidates for meniscal repair is the timeline for returning to sport. While meniscal repair aims to preserve joint function in the long term and avoid the known deleterious effects of meniscectomy, it generally requires an extended rehabilitation timeline [1,4,15,24]. Our findings align with the existing literature, demonstrating an RTS rate of 46 (86.8%), with 35 (76.1%) of these athletes representing 35 (66%) of the total cohort who resumed their pre-injury Tegner scores [25,26]. This rate falls within the upper threshold of 80%-95% typically reported following meniscal repair and is comparable to the 90% reported in a systematic review of nearly 900 patients by Totlis et al. [27].

The 11 patients who did not return to their prior activity levels were predominantly those with lower preoperative Tegner scores (≤5), suggesting greater recovery challenges in this subgroup. Conversely, the cohort with higher baseline activity levels demonstrated improved outcomes, which emphasizes the value of preoperative functional status in predicting postoperative recovery.

The average time to resume running and training was 6.1 months (median: 4.5 months), with 26 (49.1%) patients resuming activities between three and six months after surgery. This timeline is consistent with the traditionally reported 4- to 6-month interval for isolated meniscal repairs [7,27-31]. The slightly higher mean than the median reflects the heterogeneity of our cohort, with some patients requiring rehabilitation beyond nine months.

Several studies have systematically investigated factors influencing meniscal clinical healing and RTS outcomes [32-34]. In our cohort, among demographic variables, age demonstrated a selective effect: patients under 20 years achieved significantly higher postoperative Tegner scores (p = 0.020) than older patients. However, this finding must be interpreted with caution, given the small sample size of this subgroup (n = 5), which limits statistical power and increases the risk of a false positive result. IKDC functional scores remained unaffected by age. Sex did not considerably influence either outcome measure, consistent with findings by Kubiak and Fabiś [35] and others [32,36], who similarly reported no significant relationships between age, gender, body mass index (BMI), and clinical scores.

However, the literature offers divergent evidence on age as a prognostic factor. Dilworth Cannon and Vittori [37] reported an 87% clinical success rate in patients older than 40, and Hupperich et al. [33] documented superior healing rates in patients older than 39 (91.7%) compared with younger patients (53.8%), with better functional outcomes in women. These seemingly paradoxical findings may reflect that older or female patients typically engage in lower-intensity sports activities, which reduce biomechanical stress on the repair site and may compensate for age-related deficits in healing.

Smoking status emerged as a significant prognostic indicator, with smokers demonstrating substantially lower postoperative IKDC scores (68.0 ± 12.4 versus 78.1 ± 11.2 in non-smokers; p = 0.020), reflecting tobacco's deleterious effects on tissue healing. Tegner scores remained unaffected by smoking status, suggesting that while smoking compromises functional quality through persistent symptoms and pain, it does not necessarily preclude RTS within the cohort, a finding corroborated by Zabrzyński et al. [38].

Preoperative functional status was a powerful predictor of outcomes. Patients with low preoperative IKDC scores (<40) had significantly lower final IKDC scores (mean, 70.0 ± 13.2; p = 0.015) and Tegner scores (mean, 5.65 ± 2.4; p = 0.021), indicating that severe preoperative functional deterioration limits recovery potential. Similarly, preoperative Tegner scores stratified functional outcomes: patients with baseline scores exceeding 5 achieved markedly higher final Tegner scores (7.49 ± 1.5 versus 6.07 ± 2.2; p = 0.031). These findings underscore that preoperative activity levels are a robust predictor of postoperative functional outcomes, consistent with the conclusion of Logan et al. [14]. Most elite athletes can return to their pre-injury performance levels.

Regarding lesion characteristics, few studies directly compare outcomes of meniscal location. Consistent with Nakayama et al. and Moses et al. [29,39] and other comparative trials [33,40,41], no significant difference in healing rates or functional scores (Lysholm, Tegner, IKDC, KOOS) was observed between medial and lateral meniscus repairs in our cohort, even for bucket-handle tears. However, the meta-analysis by Schweizer et al. [34], integrating data from 51 studies (3,931 repaired menisci followed for 2-5 years), identified a statistically significant advantage for lateral repairs, with notably lower failure rates compared to medial repairs (6.1% versus 10.8%; p = 0.031). The authors attributed this advantage to the lateral meniscus's greater vascularity.

Circumferential location within the repair zones did not significantly correlate with functional scores, although zone 1 showed a marginally higher mean. While classical recommendations restrict repair to red-red or red-white zones, several authors have demonstrated success in avascular (white-white) zones, particularly by stimulating local bleeding to preserve meniscal tissue [35,42-46].

Lesion morphology substantially influenced outcomes. Patients with simple vertical or longitudinal lesions yielded optimal postoperative scores (mean IKDC: 81.0 ± 9.5, p = 0.040; mean Tegner: 6.84 ±1.4, p = 0.010), whereas those with complex lesions demonstrated the poorest functional outcomes (mean IKDC: 63.4 ± 14.0; mean Tegner: 5.07 ± 2.2). This dichotomy is supported by the literature, which reports inferior outcomes and healing rates for extensive or complex lesions, including bucket-handle, radial, horizontal, and flap tears that require multiple sutures to achieve stable fixation [18,30,33,36,47-50]. For example, Dilworth Cannon and Vittori [37] reported 90% healing for longitudinal lesions under 2 cm but only 50% for lesions exceeding 4 cm.

The literature consistently demonstrates superior meniscal healing outcomes when repair is combined with anterior cruciate ligament reconstruction (ACLR), attributed to enhanced knee stability, which is essential for healing; favorable biological environments created by reconstruction-induced hemarthrosis and growth factors; and standardized rehabilitation protocols, especially for elite athletes [14,34,35,37,51-57]. However, this consensus is not universal: the meta-analysis by Schweizer et al. [58] found no statistically significant difference in failure rates between isolated repairs (28%) and ACLR-combined repairs (18.7%), and Pawelczyk et al. [49] similarly observed no influence of ACLR on bucket-handle tear outcomes. The systematic review by Totlis et al. [27] noted that ACL lesions did not significantly affect overall RTS rates, though rehabilitation pathways were modified.

We deliberately excluded concomitant ACL ruptures to isolate intrinsic factors that contribute to meniscal healing. Bone marrow stimulation was systematically performed to optimize the local biological environment. Our cohort achieved an encouraging RTS rate (86.8%) and a 6.1-month recovery time. These results show excellent outcomes for isolated repairs, consistent with the literature on this specific population [27-31].

Early surgical intervention significantly influenced outcomes, as repairs performed within three months achieved substantially higher mean postoperative Tegner scores than those performed beyond six months (p = 0.029). Nevertheless, preoperative IKDC scores showed no significant temporal relationship. This dissociation suggests that while meniscal repair remains functionally effective regardless of timing, surgical promptness critically influences the likelihood of returning to pre-injury sports levels. The recent meta-analysis by Van der List et al. [59], encompassing 35 studies and 3,556 patients, demonstrated a clear correlation between delay and repair success, recommending intervention preferably within 8 weeks and noting failure rates escalating from 7.2% at 3 weeks to 15.3% thereafter. Feng et al. [51] similarly reported elevated failure rates for late repairs (>6 weeks), and Kubiak and Fabiś [35] documented significantly superior Lysholm scores (p < 0.05) for early intervention within one week. Although Haklar et al. [36] found no significant difference with a three-month delay, the preponderance of evidence supports early repair of meniscal lesions, particularly complex lesions, to maximize success rates [16,33,35].

The adverse chronicity effects likely reflect progressive muscle deconditioning, tissue degradation and retraction, structural changes, and the development of secondary cartilage lesions, all of which render anatomical repair technically challenging and create a less favorable healing microenvironment [60]. Nevertheless, chronicity alone should not be considered an absolute contraindication to repair if tissue quality remains favorable and the repair meets established criteria, including non-degenerative lesion characteristics, meticulous suture site preparation, sound surgical technique, and rigorous rehabilitation [52,61,62].

Our analysis did not stratify functional outcomes by meniscal suture technique due to a methodological limitation arising from the combined use of multiple methods in 32.1% of patients. The literature comparing IO, OI, and AI techniques reveals both similarities and differences.

Regarding subjective functional outcomes, numerous studies, including meta-analyses, have demonstrated no significant differences between AI and IO methods for successful repairs [14,31,63,64], despite AI's purported advantages in speed and technical ease [63]. Failure rate data are heterogeneous; some analyses report comparable failure rates between AI and IO techniques, whereas recent studies suggest elevated AI failure rates, particularly for medial meniscus lesions in athletes [58,65,66]. Complication profiles differ substantially; the IO technique carries a higher risk of iatrogenic nerve damage [64], whereas the AI technique presents specific implant-related risks [8,31]. Finally, limited comparative data exist for the OI technique despite its demonstrated healing potential [67].

All patients followed a standardized postoperative rehabilitation protocol designed explicitly for isolated meniscal repair. The timing of full weight-bearing was a key factor: 35 patients (66%) achieved full weight-bearing at 30 days, whereas 18 (34%) required 45 days. No statistically significant differences in final functional scores were observed between these groups, indicating flexibility in this parameter.

According to Sherman et al. [56], meniscal repair rehabilitation comprises four phases: an initial month focused on pain management and protected mobilization; a second phase restoring full ROM, complete weight-bearing, and normalized gait; a third phase (beginning month 3) addressing return to recreational activities (Tegner level 6); and a fourth phase tailored for elite athlete preparation (Tegner levels 7-9).

Modern rehabilitation is evolving toward more personalized, advanced protocols that leverage new technologies, particularly for elite athletes. Virtual reality-assisted rehabilitation and remote monitoring via connected devices are emerging technologies that support accelerated rehabilitation protocols, characterized by shortened immobilization periods, earlier joint mobility recovery, and accelerated weight-bearing resumption. Evidence supporting these protocols has confirmed their safety as expedited rehabilitation approaches [24,68-71].

Meniscal healing reflects complex interactions among numerous prognostic variables, which explain the nuanced findings and occasional contradictions observed in the published literature. To accurately interpret these differences, it is essential to understand how various factors synergistically affect individual outcomes. As Wiley et al. [53] point out, deciding when to RTS after an isolated meniscal repair is a complex and multifaceted process. It depends on both clinical factors, such as the type of lesion, the repair technique, the absence of other injuries, and the quality of rehabilitation, as well as athlete-specific factors, such as the type of sport, the competition schedule, and the athlete's risk tolerance.

A postoperative healing period of 4-6 months is conventionally recommended [55,56,72], although some protocols extend to 6-9 months [26-31]. This timeframe corresponds with the median (4.5 months) and mean (6.1 months) recovery durations recorded in our cohort for the resumption of running and training activities. For RTS clearance, both objective and subjective criteria are required [25,73]: absence of pain or effusion, full ROM, preserved stability, and sufficient muscular strength, specifically quadriceps strength symmetry exceeding 85%-90% on isokinetic testing and an appropriate hamstring-to-quadriceps ratio [69,74,75]. Additionally, functional assessment using hop test batteries and psychological readiness measures, such as the Psychological Readiness to Return to Sport (PRRS) score, is a vital component of safe, evidence-based RTS protocols [68,73]. Furthermore, validated tools such as the Knee Injury and Osteoarthritis Outcome Score (KOOS)-Sport subscale, the Lysholm score, and subjective IKDC scores are essential objective measures for assessing a patient's readiness to resume athletic activities [7,25,69,73].

RTS decision-making must additionally account for lesion-specific considerations. For instance, complex lesions, such as bucket-handle tears or radial tears, typically require stricter clearance criteria and longer timelines. In contrast, patients with stable peripheral longitudinal lesions, such as stable vertical longitudinal tears, often demonstrate accelerated recovery and achieve an earlier RTS [24,26,68,70,74]. High-level athletes, particularly younger individuals, frequently demonstrate favorable short-term clinical outcomes and high RTS rates despite incomplete structural healing on postoperative imaging, underscoring the distinction between structural healing and functional recovery [7,14].

This study has limitations that warrant consideration. First, the retrospective design inherently introduces potential selection bias, as patients lost to follow-up were excluded, and recall bias, particularly regarding the precise assessment of pre-injury activity levels. Additionally, verifying strict adherence to the rehabilitation protocol is challenging in a retrospective cohort. Second, while the sample size (N = 53) was sufficient to detect significant differences in primary functional outcomes (>99% power), it remains modest, limiting statistical power for granular subgroup analyses. Third, the assessment of meniscal healing relied exclusively on clinical and subjective functional scores, without anatomical confirmation using gold-standard methods such as second-look arthroscopy or systematic postoperative MRI. Finally, as this study was conducted at a single institution, the generalizability of these findings to broader patient populations or complex menisco-ligamentous injury patterns remains to be confirmed.

Notwithstanding these limitations, several key strengths substantiate the validity of the present cohort. First, the study addresses a clinically relevant question regarding meniscal repair outcomes in young, active patients and contextualizes the findings within the contemporary literature. The 21-month follow-up period adequately captures both short- and mid-term functional trajectories. Second, the strict selection criteria, which restricted the cohort to patients with isolated meniscal injuries, created a homogeneous population, thereby maximizing internal validity and minimizing confounding from concomitant pathology. This methodological rigor was further enhanced by standardized postoperative rehabilitation protocols and the systematic use of marrow venting as an adjunctive therapy for all patients, ensuring consistent treatment delivery and biological optimization. Finally, outcome assessment benefited from the use of validated, internationally recognized scores, specifically the IKDC and Tegner scales, which provide standardized, reliable measures of functional recovery and facilitate comparisons with the published literature.

## Conclusions

Meniscal repair remains the therapeutic standard for young athletes, offering reliable short- and mid-term functional results. Practically, early surgical intervention is recommended to optimize outcomes, particularly for athletes aiming to return to a high level of performance. It is also essential to advise patients regarding the negative impact of smoking on functional recovery. When establishing a prognosis, preoperative functional status and tear morphology must be carefully considered. Future large-scale, long-term prospective studies are needed to validate these observations, understand long-term chondroprotective effects, and refine postoperative protocols. These studies should also analyze combined lesions, compare surgical techniques, and systematically correlate clinical outcomes with objective markers of meniscal healing, such as MRI or second-look arthroscopy, to better define optimal RTS criteria.
